# Prevalence, awareness and risk factors associated with Hepatitis B infection among pregnant women attending the antenatal clinic at Mbagathi District Hospital in Nairobi, Kenya

**DOI:** 10.11604/pamj.2016.24.315.9255

**Published:** 2016-08-17

**Authors:** Jacqueline Asundula Malungu Ngaira, James Kimotho, Isaac Mirigi, Saida Osman, Zipporah Ng’ang’a, Raphael Lwembe, Missiani Ochwoto

**Affiliations:** 1 Jomo Kenyatta University of Agriculture and Technology, Institute of Tropical Medicine and Infectious Diseases, Kenya; 2 Kenya Medical Research Institute; 3 Southern Eastern Kenya University, Kenya

**Keywords:** Hepatitis B, pregnant women, prevalence, risk factors

## Abstract

**Introduction:**

Hepatitis B Viral Infection (HBV) remains one of the leading cause of morbidity and mortality globally accounting for 38-53% of chronic liver diseases and about 686,000 deaths annually. The prevalence of HBV is 9-20% in Sub-Saharan Africa, and in Kenya it is 5-30% among the general population and 9.4% among pregnant women. This study was aimed at identifying the prevalence, awareness and risk factors associated with HBV infections among pregnant women attending Antenatal clinic (ANC) at Mbagathi District hospital, Nairobi.

**Methods:**

This was a cross-sectional study involving 287 pregnant women enrolled for three months (September to December 2014) from Nairobi and neighbouring counties. A structured questionnaire that captured social, demographic and explanatory variables was administered to the study participants. Blood samples were also drawn from the participants and tested for HBV using Enzyme-Linked Immunosorbent Assay (ELISA) system.

**Results:**

The study established that the prevalence of HBV infections among pregnant women attending antenatal clinic at Mbagathi District Hospital was 3.8% with highest infection rate among the 20-24 years age group. Seventy six (60.8 %) of the participants reported sexual encounters in less than a month before the interview of which 5 (7.6%) reported encounters involving other partners apart from their spouses. HBV awareness among the study participants was 12.2%. Before the interview, those with at least tertiary education (Mean =1.33, SD = 1.131), were more informed about HBV infection as compared to those with primary and secondary education (Mean = 0.63, SD = 0.722; (Mean =0.31, SD= 0.664). In regards to assessment of the risk factors; type of family (χ^²^ =19.753 df2 p<0.01), parity (χ^²^ =7.128 df2 p<0.01), History of abortions (χ^²^=9.094 df1 p<0.01), early age (11-15 years) at first sexual encounter (χ^²^ =8.185 df1 p<0.01) were significantly associated with HBV positivity.

**Conclusion:**

The prevalence of HBV infection among pregnant women attending Antenatal clinic (ANC) at Mbagathi District hospital, Nairobi was lower (3.8%) than the prevalence among pregnant women nationally (9.4%). These women also showed a low level of HBV awareness (12.2%.).

## Introduction

Chronic HBV infections remain a major public health issue world-wide despite availability of effective vaccine and potent antiviral treatments [1]. Globally HBV accounts for over 370 million chronic infection out of which 65 million individuals reside in Sub- Saharan Africa. Approximately 2 million deaths globally are attributed to HBV infections annually [2]. Global prevalence of chronic HBV infection varies widely from high (>/= 8%), intermediate (2-7%) and low prevalence (<2%). The Sub-Saharan Africa is highly endemic [3] with prevalence's varying from one geographical location to another but higher rates are found in rural areas as compared to urban areas [1]. In Kenya, recent studies reported variations in HBV prevalence as high as 13.3% among residents of informal urban settlements to 6% among HIV infected outpatient population [4–6]. While that among pregnant women was 9.4% [7]. HBV transmission in Kenya predominantly occurs in infants through vertical transmission and in school-going children and adults through horizontal and sexual transmission respectively. Heterosexual transmission accounts for an increasing proportion of HBV infections. The peak of HBV infections occurs at early school age while the second peak occurs at puberty and childbearing age [8]. HBV awareness, access to screening, vaccination and treatment has remained poor in resource limited countries due to poverty, illiteracy and lack of political will. Unawareness of ongoing infection delays diagnosis of HBV related liver disease and favor's the spread of the virus. In studies carried out to assess HBV awareness among pregnant women in Hong Kong [9], Cameroon [10], Nigeria [11] it was noted 70-80% of respondents had poor knowledge and not aware of the infection. Most studies carried out on HBV knowledge in Kenya target health care workers. The risks factors associated with HBV infection are linked to exposure to body fluids with high concentration of the virus. During pregnancy the risk factors for HBV infection vary among communities depending on the cultural practices and beliefs [10]. Major risk factors identified in studies carried out among pregnant women and women of childbearing age include, level of education, history of blood transfusion, surgery, abortions, sexual transmitted infection, higher mean parity, early sexual debut, polygamy and higher numbers of sexual partners [12–16]. Risk factors associated to HBV prevalence in our setting need to be identified. Pregnant women are vulnerable and if infected can transmit infection to infants, children, health workers during delivery as well to sexual partners [14]. This study aimed at identifying the prevalence, awareness and risk factors associated with HBV infections among pregnant women attending Antenatal clinic at Mbagathi District hospital, Nairobi.

## Methods

**Study design**: this was a cross-sectional study in which consenting pregnant women, attending antenatal clinic at Mbagathi District Hospital were recruited over a three-month period (September and December 2014). Pretested structured questionnaires were utilized to capture socio-demographic characteristics, awareness levels and risk factors associated with HBV infection. Awareness level score was computed using Likert Scale where any correct answer was given a score and the total scores rated. HBV test results were provided to the participant on her next clinic visit.

**Study site, size and population**: the Mbagathi District Hospital is a public health facility in Nairobi, Kenya, offering health care services at a subsidized cost thus access to medical services is achievably available to everyone regardless to his/her social-economical status. The sample size estimation based on 7.7% Hepatitis B a surface antigen (HBsAg) prevalence for Nairobi [7] and using Fisher et al. formula [17] was 287. At its antenatal Clinic, an average of 65 pregnant women attended scheduled clinic days (Monday and Thursday). 20 random numbers were generated using excel work sheet for each clinic day. Utilizing the daily clinic attendance register, pregnant women whose names corresponded to the random numbers were recruited and written informed consent obtained. The procedure was followed until a sample size of two hundred and eighty seven (287) pregnant women was enrolled. Excluded were women with repeated visits and those not consenting or assenting to the study. Ethical clearance (SSC 2724) was obtained from the Kenya Medical Research Institute Scientific Ethical Review Committee before the commencement of the study.

**Specimen collection and handling:** blood samples were collected, transported to KEMRI Production Laboratory, centrifuged and stored at -80°C till use.

**HBV detection by Enzyme-Linked Immunoassay:** the blood samples were screened for the presence of Hepatitis B Surface Antigen using Hepanostika^®^ HBsAg Ultra ELISA kit (Biomerieux, Netherlands).

**Data analysis:** the data collected was subjected to descriptive and inferential statistical analysis using SPSS V.20 software (SPSS Inc. Illinios, USA). The Mean, Standard Deviation and test of comparison where categorical variables were summarized as proportions and further analyzed using Chi square and Fisher´s Exact Test to assess association between the variables. Means were compared to determine the difference in HBV awareness among subjects. Test of association using Logistic Regression was done to describe the relationship between the predictor variables (risk factors for HBV infection found to be statistically significant) and the outcome variable (HBsAg). The P value < 0.05 was considered significant.

## Results

**Socio-demographic characteristics of the pregnant women:** the study participant's ages ranged from 16 to 49 years with the mean age of 26.7± 5.5 years. More than half of the women were employed 166 (57.8%) while 122 (42.2%) were house wives. Majority of the women had classroom-based education 284 (98.1%). 252 (87.8%) women were married, 9 (3.1%) were cohabiting with a male partners while 26 (9.1%) were single. Participants were almost equally distributed among multigravidae 153 (53.3%) and primigravidae 134 (46.6%) gravidity with mean gestation period of 23.9 ±9 weeks (Table 1).

**Table 1:** Social demographic characteristic of the pregnant women

Characteristic	Number (n=287)	Number (%) HBsAg(+)
**Age Years**		
15-19	17(5.9%)	(0%)
20-24	91(31.7%)	4(4.4%)
25-29	102(35.5%)	2(2.1%)
30-34	50(17.4%)	2(4%)
35-39	21(7.3%)	2(9.5%)
40-44	5(1.7%)	1(20%)
45-49	1(0.3%)	
**Education level**		
No formal Education	3 (1.0%)	0%
Primary	81(28.2%)	5(6.2%)
Secondary	128(44.6%)	5(3.9%)
Higher	75(26.1%)	1(1.3%)
**Occupation**		
Employed formal sector	32(11.1%)	0%
Employed in formal sector	134(46.7%)	4(2.9%)
Not employed	121(42.2%)	7(5.5%)
**Marital status**		
Married	243(84.7%)	11(4.5%)
Cohabiting	9(3.1%)	0
Single	35(12.2%)	0
**Gravidity**		
Primagravida	134(46.6%)	
Multigravida	153(53.3%)	8(5.5%)
**Pregnancy stage**		
1st Trimester	41(14.3%)	3(7.3%)
2nd Trimester	93(32.4%)	4(4.3%)
3rd Trimester	153(53.3%)	4(2.6%)

* Mean age =26.7 SD=±5.5, Mean gestation period = 23.9 weeks SD=± 9

**Overall HBV prevalence**: eleven (3.8%) of the study participants tested positive for HBsAg. The prevalence was highest in the age group of 20-24 years,(4.3%). The highest prevalence was observed in multigravidae and housewives 8 (5.8%) and 7 (5.5% respectively) ( Table 1).

**HBV prevalence among participants with early age at first sexual encounter:** one hundred and forty four (50.2%) participants provided age at sexual debut; 19 (13.9%) of participants had their sexual debut between 11-15 years of age. Among them 4 (21%) were detected positive for HBsAg.

**Risky sexual behaviour among pregnant women:** the last sexual encounter with their respective partners was assessed. 125 (44%) of the participants responded out of which 76 (60.8 %) had sexual encounter in less than a month before their response to the questionnaire. Majority 48 (63%) were within age groups of 20 to 29 years. Among those who were assessed, 7 (5.6%) reported encounters involving other partners apart from their spouses. Out of which 5 were married for below 5 years and with less than 3 children.

**Awareness of Hepatitis B among pregnant women:** HBV awareness among the participants was 12.2%. Women with limited information were 108 (37.6%), while 144 ( 50.2%) had not heard about HBV infection before interview. One way ANOVA was used to compare differences in awareness among those who had attained primary, secondary and tertiary education. The results revealed statistical difference across the levels F (3, 282) =20.970, p < 001. Post-hoc Games- Howell tests revealed statistically significant differences between the participants that had attained tertiary education (Mean =1.33, SD = 1.131), and those with secondary (Mean = 0.63, SD = 0.722) and primary levels of education (Mean =0.31, SD= 0.664). Participants that had attained primary and secondary levels of education reported significantly limited awareness on HBV infection. HBV immunization was 10 (3.5%) among the enrolled subjects with results indicating difference in service uptake in relation to education attainment (F (3, 282) = 7.826, p <0.01). Post-hoc Games-Howell tests indicated significant difference between tertiary level of education (Mean =1.88, SD=0.327) and secondary level (Mean=1.99, SD= 0.088) attainment.

**HBsAg positivity and associated risk factors:** the risk factors assessed but not statistically significant included education level, history of abortions, Sexual Transmitted Infection (STI), surgery and blood transfusion. Those associated with HBsAg positivity were; type of family (χ²=19.753 df2 p<0.01), parity (χ² =7.128 df2 p<0.01), History of abortions (χ²=9.094 df1 p<0.01), and early age (11-15 years) at first sexual encounter (χ² =8.185 df1 p<0.01) (**Table 2**). Significant variables were included in a Stepwise multivariate model of logistic regression, none of them were found to be predictors of HBV infection among the participants.

**Table 2:** Association between HBsAg and predisposing risk factors

Risk Factors	HBsAg (+) (%) N=11	HBsAg (-) (%) N=276	Chi Square X2	P value
**Education level**				0.359
No formol education	0	3(100%)		
Primary	5(45.5%)	76(93.8%)		
Secondary	5(45.5%)	123(96.1%)		
Higher	1(9%	74(98.7%)		
**Type of family**			19.753	0.002*
Monogamous	7(3.0%)	228(97%)		
Polygamous	4(23.5%)	13(76.5%)		
Single	0	35(100%)		
**Parity**			7.128	0.002 *
0	3(2.2%)	131(97.8%		
<3	6(4.2%)	136(95.8%)		
>3	2(18.2%)	9(81.8%)		
**History of abortions**			9.094	0.003*
Yes	6(12.5%)	42(87.5%)		
No	5(2.09%)	234(97.9%)		
**History of STI**				
Yes	1(9.1%)	12(4.3%)	0.550	0.458
No	10(90.9%)	264(95.7%)		
**History of Surgery**				
Yes	0	36(13%)	0.667	0.414
No	11(100%)	240(87%)		
**History of blood transfusion**				
Yes	0	13 (4.7%)	0.543	0.461
No	11(100%)	263(95.3%)		
				
				
				

* Level of significance = 0.05

## Discussion

The HBsAg prevalence rate of 3.8% was found among enrolled participants indicating intermediate endemicity. Similar studies in Abuja Nigeria [11] and Bahir Ethiopia studies [18] had reported the same prevalence while studies in Tanzania [19] and Ado Ekit, Southwest Nigeria [20] had found a prevalence of 3.9%. The finding is also in close with previous studies done in Jimma Ethiopia 3.7% [15] and 4.1% Jazan region Saudi Arabia [12]. Higher prevalences have been reported in the same population in Northern Uganda 11.8% [21], 8.3% among pregnant women in low resource setting in Nigeria [14] 7.7% Yaoundé Cameroon [22], 6.9% Maiduguri Nigeria [23], 10.2% rural district far North region of Cameroon [24], 5.9% Congo [25] and 5.6% Sudan [26]. Variation in prevalences among pregnant women observed may be attributed to difference in geographical setting and study methodology adapted by the authors. Prevalence in this study is lower than the national prevalence among similar study subjects of 9.4% and that of Nairobi (7.7%) [7]. Contrarily, higher rates of HBV (6 -27.6%) have been reported from studies carried out in Nairobi among HIV infected outpatient population [6, 27, 28] and informal urban settlements residents (13.3%) [4] indicating HBV infection is well defined in high risk groups. Control of HBV infection in high risk groups is paramount as they may bridge the infection to the general population where herd immunity is limited. This has led to the rise of HBV being reported from unpublished blood donor case reports [29]. The low prevalence does not suggest absence of risky behavior among pregnant women. This study also established presence of extra marital relationships among pregnant women in monogamous marriages.

In this study majority of the women were sexually active thus predisposing them to risk of acquiring HBV infection [30, 31]. A higher rate of HBsAg was detected in age group 20-24 years (4.3%) which is the second peak of HBV infection in Kenya [8]). This findings agrees with studies among pregnant women carried out by Ajayi et al [23], Eke et al [14] Bassey et al [11] but differ with those obtained from Fomolu et al [22] study which reported higher positivity rate among age group 25-29. Although HbsAg positivity was found in all age groups, the finding supports the role of sexual transmission in HBV infection since none of the HBsAg detected cases had been exposed to blood transfusion and surgical procedures. Income is noted to be inversely associated with HBV infection. Similar trend was found in this study where higher positivity 5.5% was detected among unemployed pregnant women. This is similar to study by Ikeako et al in west Nigeria which indicated that unemployed pregnant women formed bulk of positive cases [31]. This may be explained by the low economic status initiating women to multiple sexual partnerships and unprotected sex thus making them vulnerable to STIs [21]. HBsAg positivity is reported to decrease with increase in education level [32, 33]. This did not tally with our finding where HBV positivity was equally distributed among Primary and secondary levels of education indicating education attained did not influence HBV prevalence. This may be due to limited /scarcity of focused HBV infection advocacy material and awareness within the community.

In relation to marital status there was no statistical difference between married and single pregnant women in relation to HBV positivity although all positive detected cases were married suggesting that pregnancy is not a risk factor to HBV infection. Similar results were reported from Abia state, Niger delta Nigeria [33]. Higher education attainment had a strong association with HBV infection awareness and immunization uptake. This is similar with findings by Noreen et al [34] in Punjab Pakistan and Ibadan Nigeria study [11]. This indicates that access to HBV health education is limited to higher education institutions. Women play vital roles as mothers as well as care givers to households. As per Kenya AIDS indicator survey 2012, uptake of antenatal care services in Nairobi was reported at 99.1% where 55.1% of women reported to have been tested for HIV at ANC facilities country wide. This suggests that pregnant women are easily accessible; increasing awareness in this population will go forth in rolling out HBV prevention programs in Kenya.

## Conclusion

Our study suggests a low prevalence of HBV infection among pregnant women. Occurrence of HBV infection in this population was associated with women being sexual active at an early age, history of abortions, parity and type of marital relationship (polygamous or monogamous). The findings also indicated poor HBV awareness among the subjects.

### What is known about this topic

- Hepatitis B is endemic (9.3%) in pregnant women in Kenya (Okoth et al, 2006);
- HBV infection among pregnant women reflects the same pattern as the general population (Kramvis et al., 2007);
- Risk factors to HBV infection in pregnant women varies from vary among communities depending on the cultural practices and beliefs (AA Frambo et al., 2014).

### What this study adds

- The study provides current data on HBV prevalence among pregnant women;
- The study identifies the risk factors associate with HBV infection among pregnant women in Kenya;
- The study compares if HBV prevalence among pregnant women is similar with previous study carried out in Kenya among blood donors, high risk groups and outpatient population.
